# Correlation of elevated serum uric acid with coronary artery disease in Xinjiang, China: A retrospective case-control study

**DOI:** 10.1097/MD.0000000000033256

**Published:** 2023-03-31

**Authors:** Hua-Yin Li, Hong-Yu Ji, Gulinigaer Maimaitituersun, Yi-Tong Ma, Zhen-Yan Fu

**Affiliations:** a Department of Cardiology, First Affiliated Hospital of Xinjiang Medical University, Xinjiang, China; b Xinjiang Key Laboratory of Cardiovascular Disease Research, Xinjiang, China.

**Keywords:** cardiovascular risk factors, coronary artery disease, Gensini score, serum uric acid

## Abstract

Elevated serum uric acid (SUA) levels are associated with coronary artery disease (CAD). However, whether this association is independent of traditional cardiovascular risk factors remains controversial. Our study aimed to determine the concentration of SUA in the presence and severity of CAD in multi-ethnic patients in Xinjiang, China. For this study, 412 consecutive patients with percutaneous coronary intervention (PCI) and 845 individuals with normal coronary angiograms were included in the study. CAD severity was evaluated using the Gensini score index. The SUA concentrations and the levels of various cardiometabolic risk factors were investigated. We assessed the relationship between SUA levels and other cardiometabolic risk factors. Logistic regression was used to evaluate risk factors for PCI patients. SUA levels were significantly elevated in PCI patients compared to those in control subjects (*P* < .01). With increased UA levels, we found that the risk factors for CAD increased. SUA concentration had a significant positive relationship with total cholesterol (*P* < .01), triglycerides (*P* < .01), low-density lipoprotein cholesterol (*P* < .01), and creatinine (*P* < .01) in both sexes. In the PCI group, there was no significant correlation between UA levels. SUA levels are not an independent risk factor for CAD. It can be concluded that in Xinjiang, China, SUA is related to multiple risk factors for CAD, but not related to the severity of CAD.

## 1. Introduction

The relationship between elevated serum uric acid (SUA) and coronary artery disease (CAD) and coronary atherosclerosis has always been a hot research topic. A large body of literature shows that SUA levels are related to cardiovascular risk factors such as age, male sex, hypertension, dyslipidemia, obesity, insulin resistance, and diabetes.^[1,2]^ The potential role of chronic hyperuricemia as an independent risk factor has become an important issue in determining appropriate prevention strategies.^[3]^

Uric acid is the final product of purine metabolism in apes and humans. It is mainly regulated by xanthine oxygen reductase, which converts hypoxanthine to xanthine and xanthine to uric acid. Intake of alcohol, red meat, seafood, beans, and other foods can increase uric acid levels in vitro.^[4,5]^ Compared with genetic variation, diet can explain fewer differences in SUA levels. Although experimental studies have shown that hyperuricemia causes endothelial dysfunction by increasing inflammation and oxidative stress, it has not yet been determined whether SUA is a marker of CAD, a risk factor, or a potential treatment in clinical practice.^[6]^

However, recent evidence supports the idea that hyperuricemia can be an important marker or independent risk factor for ischemic heart disease. Several studies have demonstrated a significant correlation between SUA levels and coronary atherosclerosis. Fromonot et al found that patients with confirmed coronary heart disease (CHD) had higher SUA levels than those who did not. They also found that patients with acute coronary syndrome had higher SUA levels than patients with stable CHD.^[7]^ Deveci et al used the Gensini score and found that the severity of ischemic heart disease was strongly related to the level of SUA.^[8]^ In contrast, another conclusion is that the level of SUA is related to the presence of CAD, but not to severity.^[9]^ Duran et al found that hyperuricemia was positively correlated with angiography severity of CAD.^[10]^ Barbieri et al found that SUA levels were significantly higher in men than in women, while high UA levels were only associated with severe CAD in women.^[11]^ However, there may be an association between SUA levels and the presence and severity of CAD in multi-ethnic patients in Xinjiang, China. The study aimed to investigate the relationship between SUA levels and the presence and severity of CAD in multi-ethnic patients in Xinjiang, China.

## 2. Materials and methods

### 2.1. Sample design

This was a retrospective case-control study. We recruited patients who were hospitalized in the First Affiliated Hospital of Xinjiang Medical University between 2015 and 2018. We included 1257 participants (male: 682; female: 575; Han [547]), Uyghur (503), and Kazakh (207). Patients were between 30 and 85 years old, mean ± standard deviation age of 53.85 ± 9.97 years (male 52.71 ± 9.33 years; female 55.05 ± 10.47 years). Among them, 412 patients with percutaneous coronary intervention (PCI) were defined as the case group, and 845 normal coronary angiographies were defined as the control group. All the patients were clinically stable and continued to take CHD drugs during the study period. All patient and control groups provided written informed consent. Exclusion criteria were the presence of tumor disease, recent major surgery, diuretics, accompanying inflammatory diseases, such as infection and autoimmune disease; liver or kidney disease; patients who had undergone coronary artery bypass grafting; and patients with valvular disease or myocardial or pericardial diseases were also excluded. Blood samples were obtained, including SUA concentration, creatinine, total cholesterol (TC), triglycerides (TG), high-density lipoprotein cholesterol (HDL-C), low-density lipoprotein cholesterol (LDL-C), and fasting blood glucose. Our study was approved by the Ethics Committee of the First Affiliated Hospital of Xinjiang Medical University and conducted in accordance with the standards of the Declaration of Helsinki. Written informed consent was obtained from all participants.

### 2.2. Baseline definitions and measurements

Hypertension was defined as self-reported use of antihypertensive drugs or in the past 2 weeks having an average systolic blood pressure (SBP) ≥ 140 mm Hg, or an average diastolic blood pressure (DBP) ≥ 90 mm Hg. Diabetes was defined as an FPG level ≥ 7.0 mmol/L or previous diabetes diagnosis or use of diabetes drugs. Smoking was defined as the patient’s current smoking status. TC concentration ≥ 6.22 mmol/L (240 mg/dL) was defined as hypercholesterolemia. Triglyceride concentration ≥ 2.26 mmol/L (200 mg/dL) was hypertriglyceridemia. LDL-C concentration ≥ 4.14 mmol/L (160 mg/dL) was defined as high LDL cholesterol. HDL-C concentration ≤ 1.04 mmol/L (40 mg/dL) was defined as low HDL cholesterol. Dyslipidemia is defined as one of the 4 above-mentioned dyslipidemias or the self-reported use of anti-hyperlipidemia drugs.^[12]^ After 12 hours of fasting, the patient’s peripheral venous blood was collected for routine laboratory parameter evaluation. SUA, TC, TG, HDL-C, LDL-C, fast glucose, and creatinine concentrations were measured by a clinical laboratory department biochemical analyzer of the First Affiliated Hospital of Xinjiang Medical University (Dimension AR/AVL Clinical Chemistry System, Newark, NJ). Coronary angiography was performed and interpreted by an interventional cardiologist with >5 years of work experience. The modified Gensini score was calculated using the scoring model defined by Gensini et al.^[13]^ To avoid observational bias, the Gensini score was calculated by 3 independent cardiologists, and the average value of the 3 experts was combined for the final analysis.

### 2.3. Statistical analysis

All statistical analyses were performed using the Social Science Statistical Software Package (SPSS) software (version 25.0; SPSS Inc., Chicago, IL). Continuous variables were expressed as mean ± standard deviation, and an independent sample *t*-test was used for comparison. Categorical variables were expressed as numbers and percentages, and the chi-square test was used for analysis. Spearman correlation analysis was used to detect the relationship between serum SUA levels and other variables. Logistic regression analysis was used to investigate the relationship between the SUA and CAD. We established 3 logistic regression models: model 1, adjusted for SUA; model 2, adjusted for model 1 + age, ethnic group (Han, Uygur, Kazakh), BMI, sex (male, female); model 3, adjusted for model 2 + hypertension, diabetes, smoking, and dyslipidemia. All statistical tests were 2-sided tests, and statistical significance was set at *P* < .05.

## 3. Results

### 3.1. Baseline characteristics of participants

Table 1 demonstrates the differences in clinical characteristics between male and female participants. Compared with the controls, the patients had a higher age, DBP, SUA, LDL-C, and fasting glucose. The patients had a high rate of hypertension, diabetes, and smoking. Table 2 shows the differences in the clinical characteristics of female study participants. In women, compared with the controls, the patients had higher age, BMI, SBP, creatinine, TG, and fasting glucose. The patients had a high rate of hypertension, diabetes, and smoking.

**Table 1:** Baseline characteristics of male and female.

	Male				Female			
	Overall (682)	Controls (417)	Case (265)	*P*	Overall (575)	Controls (428)	Case (147)	*P*
Age (yr)	52.71 ± 9.33	51.28 ± 9.07	55.10 ± 9.31	<.01	55.05 ± 10.47	52.44 ± 9.56	62.84 ± 9.12	<.01
Ethnicity	682	417	265	.06	575	428	147	.74
Han (n, %)	275 (40.3)	173 (41.5)	102 (38.5)		272 (47.3)	203 (47.4)	69 (46.9)	
Uygur (n, %)	262 (38.4)	175 (42)	87 (32.8)		241 (41.9)	176 (47.1)	65 (44.2)	
Kazakh (n, %)	145 (21.3)	69 (16)	76 (28.7)		62 (10.8)	49 (11.5)	13 (8.9)	
BMI	26.85 ± 4.57	26.72 ± 4.11	27.06 ± 5.22	.37	25.77 ± 4.01	25.60 ± 4.02	26.27 ± 3.95	.09
Abdominal circumference	89.17 ± 37.14	91.68 ± 44.57	85.27 ± 20.55	.07	82.75 ± 12.39	82.16 ± 12.79	84.30 ± 11.19	.15
SBP, mm Hg	137.14 ± 53.48	134.37 ± 47.92	141.40 ± 60.92	.10	135.51 ± 29.7	130.69 ± 27.00	149.52 ± 32.98	<.01
DBP, mm Hg	81.95 ± 18.31	80.71 ± 17.40	83.86 ± 19.51	.03	82.62 ± 33.40	80.99 ± 36.84	87.38 ± 19.65	<.01
Creatinine	89.17 ± 37.14	78.52 ± 4.94	82.06 ± 59.92	.41	60.20 ± 24.87	58.83 ± 12.87	64.22 ± 43.99	.02
SUA, µmol/L	334.27 ± 79.97	333.08 ± 79.11	336.11 ± 81.41	<.01	267.74 ± 73.98	262.47 ± 70.20	283.29 ± 82.47	<.01
TC, mmol/L	3.70 ± 0.96	3.68 ± 0.92	3.73 ± 1.03	.58	4.04 ± 1.10	4.01 ± 1.10	4.12 ± 1.09	.30
TG, mmol/L	1.95 ± 2.24	1.97 ± 2.64	1.91 ± 1.31	.72	1.60 ± 1.09	1.47 ± 0.98	1.98 ± 1.32	<.01
HDL-C, mmol/L	1.05 ± 0.64	1.09 ± 0.67	0.99 ± 0.59	<.05	1.17 ± 0.37	1.22 ± 0.38	1.01 ± 0.31	<.01
LDL-C, mmol/L	2.37 ± 0.77	2.31 ± 0.73	2.46 ± 0.82	.02	2.55 ± 0.83	2.52 ± 0.83	2.63 ± 0.82	.16
Fast glucose, mmol/L	5.81 ± 2.65	5.43 ± 2.40	6.40 ± 2.92	<.01	5.88 ± 3.01	5.53 ± 2.75	6.90 ± 3.47	<.01
Hypertension (n, %)	273 (40)	146 (35)	127 (47.9)	<.01	165 (28.7)	108 (25.2)	57 (38.8)	<.01
Diabetes (n, %)	97 (14.2)	37 (8.9)	60 (22.6)	<.01	60 (10.4)	35 (8.2)	25 (17)	<.01
Smoking (n, %)	385 (56.5)	224 (53.7)	161 (60.8)	<.01	151 (26.3)	91 (21.3)	60 (40.8)	<.01

BMI = body mass index, DBP = diastolic blood pressure, HDL-C = high-density lipoprotein cholesterol, LDL-C = low-density lipoprotein cholesterol, SBP = systolic blood pressure, SUA = serum uric acid, TC = total cholesterol, TG = triglyceride.

**Table 2:** Association of uric acid quartiles and baseline characteristics of male and female.

	Male					Female				
	Group I (n = 76)	Group II (n = 134)	Group III (n = 189)	Group IV (n = 249)	*P*	Group I (n = 234)	Group II (n = 161)	Group III (n = 112)	Group IV (n = 56)	*P*
Age (yr)	54.87 ± 8.87	53.08 ± 8.71	52.00 ± 9.33	52.33 ± 9.62	.32	52.40 ± 11.41	55.09 ± 9.72	57.35 ± 8.60	59.66 ± 8.98	<.01
BMI (kg/m^2^)	25.63 ± 5.96	25.69 ± 4.44	27.15 ± 4.87±	27.46 ± 3.78	<.01	25.06 ± 4.02	25.76 ± 4.16	26.67 ± 3.85	26.95 ± 3.52	<.01
Abdominal circumference	87.87 ± 17.72	88.57 ± 14.91	93.86 ± 63.09	85.51 ± 16.56	.45	82.39 ± 12.03	80.34 ± 11.62	85.21 ± 13.72	87.84 ± 11.90	<.01
TC, mmol/L	3.83 ± 0.95	3.50 ± 0.85	3.69 ± 0.94	3.83 ± 1.01	.01	3.92 ± 1.03	4.03 ± 1.13	4.13 ± 1.00	4.39 ± 1.21	.03
TG, mmol/L	1.55 ± 0.85	1.49 ± 0.87	1.74 ± 1.26	2.05 ± 1.53	<.01	1.37 ± 0.94	1.65 ± 1.20	1.79 ± 0.98	2.09 ± 1.35	<.01
HDL-C, mmol/L	1.12 ± 1.06	1.06 ± 0.32	1.07 ± 0.68	1.00 ± 0.59	.45	1.18 ± 0.32	1.20 ± 0.34	1.14 ± 0.33	1.10 ± 0.63	.31
LDL-C, mmol/L	2.43 ± 0.71	2.23 ± 0.71	2.35 ± 0.75	2.47 ± 0.81	.03	2.45 ± 0.79	2.48 ± 0.78	2.68 ± 0.85	2.80 ± 0.98	<.01
Fast glucose, mmol/L	7.00 ± 3.93	5.72 ± 2.99	5.55 ± 2.31	5.63 ± 2.05	<.01	5.81 ± 2.67	5.60 ± 2.40	6.11 ± 4.10	6.84 ± 3.14	.05
Creatinine	68.53 ± 17.19	70.28 ± 14.76	81.50 ± 68.71	85.64 ± 58.96	.01	55.64 ± 12.21	59.89 ± 11.91	68.62 ± 47.90	65.27 ± 18.09	<.01
SUA, µmol/L	210.06 ± 42.67	272.79 ± 14.03	324.73 ± 16.21	413.19 ± 52.63	<.01	204.46 ± 33.31	269.82 ± 13.45	320.63 ± 17.52	421.16 ± 59.55	<.01
Hypertension (n, %)	24 (31.6)	54 (40.3)	82 (42.3)	103 (41.4)	.36	56 (23.9)	56 (34.8)	33 (29.5)	18 (32.1)	.12
Diabetes (n, %)	20 (26.3)	20 (14.9)	24 (12.7)	30 (12.0)	.02	29 (12.4)	17 (10.6)	6 (5.4)	7 (12.5)	.23
Smoking (n, %)	40 (52.6)	66 (49.3)	109 (57.7)	146 (58.6)	.30	54 (23.1)	42 (26.1)	32 (28.6)	18 (32.1)	.47

BMI = body mass index, HDL-C = high-density lipoprotein cholesterol, LDL-C = low-density lipoprotein cholesterol, SUA = serum uric acid, TC = total cholesterol, TG = triglyceride.

### 3.2. Association of uric acid quartiles and baseline characteristics

Table 2 shows the SUA quartiles used in this study for men and women: in men, Q1, <282 μmol/L, Q2, 283–329 μmol/L, Q3, 330–385 μmol/L, and Q4, >386 μmol/L. We found that increased SUA levels were associated with increased BMI, TC, TG, LDL-C, fasting glucose, and creatinine. But in females (Q1, <218 μmol/L, Q2, 219–259 μmol/L, Q3, 260–303 μmol/L, and Q4, >304 μmol/L), we found that increased SUA levels were associated with increased age, BMI, abdominal circumference, TC, TG, and LDL-C. Females were slightly different from males. However, with increased uric acid levels, we found that the risk factors for CAD increased.

### 3.3. Correlation of SUA concentration with CAD cardiometabolic risk factors

In males, Spearman correlation analysis revealed that SUA concentration had a significant positive relationship with BMI (*R* = 0.18, *P* < .01), TC (*R* = 0.11, *P* < .01), TG (*R* = 0.22, *P* < .01), LDL-C (*R* = 0.10, *P* < .01), and creatinine (*R* = 0.33, *P* < .01). However, SUA also showed a significant negative correlation with age (*r* = −0.10, *P* < .05), ethnic group (*r* = −0.15, *P* < .01), and HDL-C (*r* = −0.15, *P* < .01). In females, SUA concentration was positively correlated with age (*R* = 0.24, *P* < .01), SBP (*R* = 0.16, *P* < .01), DBP (*R* = 0.16, *P* < .01), BMI (*R* = 0.20, *P* < .01), TC (*R* = 0.11, *P* < .01), TG (*R* = 0.28, *P* < .01), LDL-C (*R* = 0.10, *P* = .02), fasting glucose (*R* = 0.11, *P* < .01), and creatinine (*R* = 0.27, *P* < .01). In contrast, SUA was negatively correlated with HDL-C levels (*r* = −0.09, *P* < .04) (Table 3).

**Table 3:** Spearman correlation of SUA with various cardiometabolic risk factor levels.

	SUA (male)		SUA (female)	
Parameters	*r*	*P*	*r*	*P*
Age	−0.10	<.05*	0.24	<.01
Ethnic group	−0.15	<.01*	−0.05	.23
SBP	−0.02	.65	0.16	<.01
DBP	0.04	.36	0.16	<.01
BMI (kg/m^2^)	0.18	<.01*	0.20	<.01
Abdominal circumference	−0.06	.26	0.09	.09
TC, mmol/L	0.11	<.01*	0.11	<.01
TG, mmol/L	0.22	<.01*	0.28	<.01
HDL-C, mmol/L	−0.15	<.01*	−0.09	.04
LDL-C, mmol/L	0.10	<.01*	0.10	.02
Fast glucose, mmol/L	−0.003	.93*	0.11	<.01
Creatinine	0.33	<.01*	0.27	<.01

*P < 0.05. BMI = body mass index, DBP = diastolic blood pressure, HDL-C = high-density lipoprotein cholesterol, LDL-C = low-density lipoprotein cholesterol, SBP = systolic blood pressure, SUA = elevated serum uric acid, TC = total cholesterol, TG = triglyceride.

### 3.4. Correlation of cardiometabolic indicators levels with different CAD severity

In the subgroup study of females, on the severity of CAD, the age, LDL-C showed significant differences with the gradual increase of Gensini score, but there was no significant change in males. There were no significant differences in BMI, abdominal circumference, TC, TG, HDL-C, fasting glucose, creatinine, and the prevalence of diabetes, hypertension, and smoking among different subgroups in PCI patients (*P* > .05) (Table 4).

**Table 4:** SUA and other cardiometabolic indicators levels in different coronary artery disease severity subgroups evaluated by Gensini scoring system.

	Male					Female				
	Group I (n = 51)	Group II (n = 68)	Group III (n = 68)	Group IV (n = 69)	*P*	Group I (n = 49)	Group II (n = 34)	Group III (n = 35)	Group IV (n = 27)	*P*
Age (yr)	54.13 ± 7.46	54.20 ± 8.26	57.23 ± 10.19	54.27 ± 9.76	.318	62.75 ± 9.23	59.16 ± 12.02	64.96 ± 6.70	63.43 ± 8.18	.207
BMI (kg/m^2^)	27.79 ± 4.27	26.83 ± 3.71	27.19 ± 6.61	26.52 ± 5.95	.638	24.89 ± 3.58	26.28 ± 2.66	26.35 ± 3.36	29.08 ± 5.54	<.001
Abdominal circumference	8.36 ± 23.19	87.03 ± 1162	83.09 ± 23.87	85.21 ± 18.76	.748	85.86 ± 12.19	83.00 ± 8.63	83.42 ± 9.08	84.27 ± 17.10	.781
TC, mmol/L	3.95 ± 1.04	3.71 ± 102	3.81 ± 1.14	3.54 ± 0.94	.206	4.11 ± 1.13	4.03 ± 1.04	4.46 ± 1.24	3.87 ± 0.87	.199
TG, mmol/L	2.26 ± .55	1.91 ± 1.58	1.85 ± 1.26	1.81 ± 0.92	.325	2.02 ± 1.45	2.10 ± 1.45	2.01 ± 1.24	1.78 ± 1.05	.822
HDL-C, mmol/L	0.97 ± 0.27	1.08 ± 0.81	0.94 ± 0.25	0.96 ± 0.76	.576	1.05 ± 0.32	0.95 ± 0.3	0.98 ± 0.29	1.06 ± 0.34	.414
LDL-C, mmol/L	2.59 ± 0.85	2.43 ± 0.76	2.54 ± 0.94	2.33 ± 0.73	.332	2.48 ± 0.85	2.50 ± 0.81	3.01 ± 0.77	3.22 ± 0.76	.026
Fast glucose, mmol/L	6.30 ± 3.92	6.03 ± 2.59	6.56 ± 2.44	6.66 ± 2.96	.614	6.50 ± 2.53	7.10 ± 3.25	6.79 ± 3.81	6.97 ± 3.37	.854
Creatinine	79.86 ± 37.06	75.00 ± 17.18	95.35 ± 109.56	77.94 ± 18.81	.212	76.19 ± 72.60	56.72 ± 13.79	58.10 ± 18.76	60.09 ± 16.80	.15
SUA, µmol/L	330.78 ± 86.11	339.06 ± 86.53	337.48 ± 77.96	334.03 ± 78.29	.952	289.38 ± 8144	261.04 ± 87.18	292.64 ± 64.46	292.90 ± 97.13	.324
Hypertension (n, %)	23 (45.1)	34 (50.0)	37 (544)	29 (42.0)	.496	9 (18.4)	20 (58.8)	13 (37.1)	14 (51.9)	.001
Diabetes (n, %)	15 (29.4)	14 (20.6)	10 (14.7)	21 (30.4)	.107	3 (6.1)	9 (26.5)	6 (17.1)	7 (25.9)	.053
Smoking (n, %)	37 (72.5)	39 (57.4)	38 (55.9)	41 (59.4)	.259	36 (73.5)	7 (20.6)	12 (34.3)	4 (14.8)	<.001
Gensini score	12.17 ± 5.50	30.84 ± 6.50	51.46 ± 6.94	91.16 ± 21.43	<.001	12.17 ± 6.53	29.65 ± 6.45	52.89 ± 7.36	84.44 ± 13.36	<.001

BMI = body mass index, HDL-C = high-density lipoprotein cholesterol, LDL-C = low-density lipoprotein cholesterol, SUA = serum uric acid, TC = total cholesterol, TG = triglyceride.

### 3.5. Association of SUA with CAD

Logistic regression analysis showed that age (OR = 1.05, 95% CI [1.02–1.08], *P* < .01), Kazakh versus Han (OR = 2.29, 95% CI [1.46–4.54], *P* = .02), hypertension (OR = 1.70, 95% CI [1.06–2.72], *P* = .03), and diabetes (OR = 2.22, 95% CI [1.21–4.09], *P* = .01) were independent risk factors for CAD in men. However, in women, logistic regression analysis showed that age (OR = 1.14, 95% CI [1.10–1.18], *P* < .01), Uygur versus Han (OR = 2.48, 95% CI [1.25–4.94], *P* = .01), hypertension (OR = 2.45, 95% CI [0.91–2.39], *P* = .02), and smoking (OR = 5.22, 95% CI [2.51–10.84], *P* < .01) were independent risk factors for CAD. In both men and women, SUA is not an independent risk factor for CAD (Table 5). After multivariate adjustment for confounding factors, SUA was still not an independent risk factor for CAD (Table 6).

**Table 5:** Logistic regression between different quartiles of uric acid level with CAD.

	CAD (male)		CAD (female)	
Variables	OR (95% CI)	*P* value	OR (95% CI)	*P* value
Age (yr)	1.05 (1.02–1.08)	<.01	1.14 (1.10–1.18)	<.01
BMI (kg/m^2^)	1.01 (0.96–1.06)	.78	1.03 (0.95–1.12)	.48
Ethnic group				
Uygur vs Han	0.89 (0.51–1.58)	.70	2.48 (1.25–4.94)	.01
Kazakh vs Han	2.29 (1.16–4.54)	.02	0.71 (0.26–1.98)	.52
Hypertension, yes vs no	1.70 (1.06–2.72)	.03	2.45 (1.45–5.26)	.02
Diabetes, yes vs no	2.22 (1.21–4.09)	.01	1.71 (0.67–4.37)	.26
Smoking, yes vs no	1.47 (0.91–2.39)	.12	5.22 (2.51–10.84)	<.01
Dyslipidemia	1.37 (0.81–2.32)	.24	1.94 (1.11–3.41)	.21
SUA (mg/dL)	1.001 (0.99–1.01)	.72	1.001 (0.99–1.01)	.45

BMI = body mass index, CAD = coronary artery disease, CI = confidence interval, OR = odds ratio, SUA = serum uric acid.

**Table 6:** Adjusted odds ratios (95% CI) for associations between CAD and SUA levels.

Model	Q1	*P*	Q2 (OR)	*P*	Q3 (OR)	*P*	Q4 (OR)	*P*
1	1	–	1.09	.63	1.42	<.05	1.69	<.01
2	1	–	0.91	.69	0.94	.81	1.17	.55
3	1	–	0.89	.76	0.84	.66	0.69	.36

Model 1: Adjusted for serum uric acid; Model 2: adjusted for model 1 + age, ethnic group, body mass index (BMI), and gender; Model 3: adjusted for model 2 + hypertension, diabetes, smoking, and dyslipidemia.

CAD = coronary artery disease, CI = confidence interval, OR = odds ratio, SUA = elevated serum uric acid.

### 3.6. Analysis of receiver operating characteristic curve

The receiver operating characteristic curve analysis showed that the best diagnostic cutoff value of SUA for CAD was 4.647, the specificity was 44.1%, the sensitivity was 68.4%, the Youden index was 0.125, and the area under the curve was 0.563 (95% CI: 0.529–0.597, *P* < .01; Table 7).

**Table 7:** Predictive value of serum uric acid (SUA) for coronary artery disease (CAD).

Cutoff	Specificity	Sensitivity	Youden index	AUC (95% CI)
4.647	44.10%	68.40%	0.125	0.563 (0.529–0.597, *P* < .01)

AUC = area under the curve, CAD = coronary artery disease, CI = confidence interval, SUA = elevated serum uric acid.

## 4. Discussion

Our study found the following: the SUA level of the PCI patient group was significantly higher than that of the control group, which excluded CHD by coronary angiography, regardless of whether it was male or female; when SUA levels increased, the risk factors for CAD also increased; there was no significant association between SUA level and the severity of CAD; hyperuricemia is not an independent risk factor for CAD in Xinjiang, China.

In our study, compared with controls, the patients had a higher age, DBP, SUA, LDL-C, fasting glucose, and a higher rate of hypertension, diabetes, and smoking in males. In females. the patients had higher age, BMI, SBP, creatinine, TG, fasting glucose, and a high rate of hypertension, diabetes, and smoking. This conclusion is similar to previous reports.^[14]^ We also found that compared with SUA levels below quartiles, higher SUA levels gradually increased the risk factors for CAD. Empar Lurbe et al^[15]^ found that with an increase in uric acid levels, there is a set of related metabolic risk factors. We used Spearman correlation analysis and found that SUA concentration had a significant positive relationship with age, BMI, blood pressure, TC, TG, LDL-C, and creatinine. Therefore, we believe that the association between SUA and CAD is more likely to stem from the close link between SUA and CAD risk factors.

We also found that when there was only SUA as a variable, the high quartile SUA group (OR = 1.69, *P* < .01) increased the risk of CAD compared to the low quartile SUA group. However, when we adjusted for other variables, the high quartile SUA group had no statistical significance in the low quartile SUA group. We speculated that SUA may not be an independent risk factor for CAD. Krishnan et al^[16]^ and De Luca et al^[17]^ also showed that there is no clear conclusion to determine whether SUA is an independent predictor of CAD. High SUA levels at admission have been independently related to hospitalization and long-term adverse outcomes, but whether it is related to poor coronary blood flow has not been determined.^[18–20]^ It is well established that UA induces inflammation of vascular endothelial cells and smooth muscle cells and intracellular oxidative stress, finally leading to endothelial dysfunction.^[21,22]^ Conversely, inflammation and oxidative stress are considered to be key factors in the development of CAD through atherosclerosis and thrombosis.^[23]^ In addition, SUA was positively correlated with arterial intima-media thickness, which is a precursor of atherosclerosis and CAD.^[24]^ In brief, it has changed the metabolism and endocrine systems. Through sympathetic activation and increased levels of inflammatory cytokines, the development of hyperuricemia may be related to the pathogenesis of CHD.

We found that SUA concentration had a significant positive relationship with TC, TG, and LDL-C, and a negative correlation with HDL-C in males and females. Minkook Son et al^[25]^ arrived at the same conclusion as our study. There are many factors associated with human hyperuricemia, including age, sex, ethnicity, genetics, and dietary factors. The Framingham Heart Study showed that baseline UA levels cannot predict future cardiovascular events, including CAD and death, after adjusting for confounding clinical factors.^[26]^ However, some studies have shown that SUA levels are significantly associated with the risk of death from CAD.^[27,28]^ These results suggest a relationship between UA and CHD through CHD risk factors.

There are some limitations to our study. First, this was a retrospective, single-center analysis with a relatively small number of patients. As this was a retrospective case-control analysis study, we cannot rule out the existence of selection bias or confounding bias, and the result restricted us from drawing causal conclusions. Our results should be interpreted carefully as a hypothesis for further research. Our study cannot provide data on the long-term effects of SUA on cardiovascular prognosis. Second, the control group selected patients with normal coronary angiography, not entirely healthy individuals. However, to investigate the relationship between UA level and CAD severity, this condition was not important.

## 5. Conclusions

Our study demonstrated that in Xinjiang, China, SUA is related to multiple risk factors for CAD, but is not related to the severity of CAD.

## Acknowledgments

We are very grateful to all the participants in this study for their enthusiastic collaboration and to the personnel from First Affiliated Hospital of Xinjiang Medical University Cardiology Center for their assistance in data collection.

## Author contributions

**Conceptualization:** Hua-Yin Li, Hong-Yu Ji, Zhen-Yan Fu.

Data curation: Hua-Yin Li.

Formal analysis: Hua-Yin Li, Gulinigaer Maimaitituersun.

Writing – original draft: Hua-Yin Li, Hong-Yu Ji.

Writing – review & editing: Hua-Yin Li, Yi-Tong Ma, Zhen-Yan Fu.
